# Complete Genome Sequence of an Atypical Dengue Virus Serotype 2 Lineage Isolated in Brazil

**DOI:** 10.1128/genomeA.00779-15

**Published:** 2015-07-16

**Authors:** Felipe Scassi Salvador, Jaime Henrique Amorim, Rubens Prince Santos Alves, Sara A. Pereira, Luis Carlos Souza Ferreira, Camila Malta Romano

**Affiliations:** a São Paulo Institute of Tropical Medicine, University of São Paulo School of Medicine, São Paulo, Brazil; b Institute of Biomedical Sciences, University of São Paulo, São Paulo, Brazil

## Abstract

Here, we report the complete polyprotein sequence of a dengue virus 2 strain isolated in Brazil. This virus belongs to the American genotype and has the ability to cause neurovirulence in immunocompetent adult mice. The data presented here may help understand the genetic determinants responsible for neurovirulence.

## GENOME ANNOUNCEMENT

Dengue virus (DENV), which is composed of four distinct serotypes (DENV1 to -4), is responsible for the most prevalent arthropod-borne viral infection of humans in tropical and subtropical countries. The mechanisms involved in dengue pathogenesis and severity of the symptoms vary according to host and viral factors. In fact, natural DENV variants may express distinct molecular and physiological features that can impact viral fitness and virulence.

We report here the near-full-length genome sequence of an American strain of DENV2 (JHA1 strain). This virus was isolated from a patient in Belém do Pará, Brazil, presenting with dengue fever (1), and this strain was observed to be neurovirulent in immunocompetent adult mice.

The partial sequencing of the envelope and nonstructural 1 (NS1) genes of the JHA1 strain showed that the virus belongs to the serotype 2 American genotype. Since the capacity to kill immunocompetent adult mice after intracranial inoculation is an atypical virulence property of this virus, we decided to determine the full-length sequence encoding the viral polyprotein.

The virus was PCR amplified using a previously described protocol (2) with three subsets of primers that covered the complete polyprotein. Fragments were sequenced using the Ion Torrent platform (Life Technologies). Reads were assembled using the genome of a DENV2 American genotype virus as a reference (accession no. JX966380).

Phylogenetic analysis using both envelope and complete genome confirmed that this virus is a DENV2 serotype of the American genotype. Nevertheless, JHA1 was the most divergent virus among all American reference genomes (*n* = 29; GenBank accession numbers HM582099 to HM582117, GQ868588, GQ868589, GQ868599, EU056811, EU0568112, FJ898449, JX966380, AY702040, and AY744147). Genetic distance was estimated for the American viruses (excluding JHA1) and also between JHA1 and other American strains. The average distance (p-distance) among American viruses (without JHA1) was 0.015, which is in accordance with the genetic distance observed for viruses belonging to the same genotype. However, JHA1 presented about 6.0% divergence (p-distance = 0.058 to 0.064) from other American viruses. Intriguingly, according to the envelope-based tree, JHA1 was very closely related to Trin53, which was isolated in 1953 from a female patient in Trinidad and Tobago (3). A pairwise comparison of Trin53 and JHA1 (1,485 bp) revealed five synonymous changes and one nonsynonymous change (p-distance = 0.033). Unfortunately, there is no available sequence other than the envelope gene of Trin53 to allow for a more detailed comparison between these two strains.

Similarities to the Trin53 virus were not restricted to the envelope gene. Similarly to Trin53, JHA1 rapidly replicates in brains of suckling mice and causes encephalitis. Nevertheless, JHA1, in contrast to Trin53, is harmful to adult immunocompetent mice.

Although with the available information, no clear conclusions can be drawn regarding the polymorphic sites that confer such neurovirulence to JHA1, a comparison with the New Guinea C (NGC) neurovirulent mouse-adapted strain revealed that both viruses have a lysine (K) at position 126, identified as a major determinant of neurovirulence in NGC (4).

In sum, we described here the complete polyprotein sequence of a DENV2 isolated in Brazil, which is atypically neurovirulent to adult immunocompetent mice and belongs to the American genotype.

### Nucleotide sequence accession numbers.

The genome described in this work has been deposited in GenBank under the accession no. JQ686088. The version described in this paper is the updated version, JQ686088.2.
